# System-level hypothesis of dopamine imbalance in early multiple sclerosis

**DOI:** 10.3389/fneur.2025.1653134

**Published:** 2025-09-25

**Authors:** Daniele Caligiore, Aurelia Schirripa, Monica Biggio

**Affiliations:** ^1^Computational and Translational Neuroscience Laboratory, Institute of Cognitive Sciences and Technologies, National Research Council (CTNLab-ISTC-CNR), Rome, Italy; ^2^AI2Life s.r.l., Innovative Start-Up, ISTC-CNR Spin-Off, Rome, Italy; ^3^Department of Neuroscience, Rehabilitation, Ophtalmology Genetics, Maternal and Child Health, DINOGMI, University of Genoa, Largo Paolo Daneo, Genoa, Italy

**Keywords:** clinically isolated syndrome, demyelination, dopamine dysregulation, multiple sclerosis, network neuroscience, neurodegeneration, neurotransmitter imbalance, optic neuritis

## Abstract

Multiple Sclerosis (MS) is a chronic autoimmune disorder of the central nervous system, with evidence suggesting that age-related brain changes may influence its progression. Clinically Isolated Syndrome (CIS) often marks an early phase of MS, with optic neuritis frequently presenting as a symptom. Despite recognition as an early indicator, the mechanisms driving optic neuritis and its contribution to MS progression remain unclear. Traditionally, immune-mediated inflammation has dominated MS research; however, emerging evidence highlights neurotransmitter dysregulation—especially involving dopamine—as a crucial factor in disease pathophysiology. The impact of dopamine imbalance on neural circuits and its role in advancing MS requires further investigation. This paper proposes a system-level, dopamine-based hypothesis to explain MS origins, focusing on early stages in CIS. Building on a review of recent literature linking dopaminergic dysfunction, neuroinflammation, and demyelination, the model suggests that optic nerve demyelination, as seen in optic neuritis, disrupts dopamine signaling, triggering a cascade of neural alterations that drive MS pathogenesis. By emphasizing dopamine role in CIS and early MS, this framework offers a novel perspective on the neurobiological mechanisms underlying the disease. This approach complements current research on neurotransmitter involvement in age-related conditions, expanding understanding of how neurotransmitter imbalances may influence MS and related disorders.

## 1 Introduction

Multiple Sclerosis (MS) is a chronic, debilitating autoimmune disorder of the central nervous system (CNS), characterized by progressive demyelination and neurodegeneration (1, 2). Similar to other neurodegenerative diseases, MS is often marked by age-related changes in brain structure and function, with disease progression commonly correlating with aging. Although MS is not explicitly categorized as an age-related disease, aging may exacerbate its underlying pathophysiology, and age-related changes in the brain may contribute to the acceleration of disease progression (3–5). Clinically Isolated Syndrome (CIS) is often an early manifestation of MS. It is a monophasic clinical episode reflecting inflammatory demyelinating event in the central Nervous System CNS (6). Its specific manifestations could interest various location, one among all is the optic nerve. Even if neuromyelitis optica spectrum disorder (NMOSDs) has been discovered to be a different pathology from MS (with potentially confounding clinical and imaging features (6) but different treatment (7, 8), the mere inflammation of the optic nerve is still the most common initial manifestation of CIS suggestive of MS (6, 9–14). Although optic neuritis serves as an early clinical marker, the mechanisms driving its onset and its contribution to MS progression remain unclear (15, 16). While immune-mediated inflammation plays a central role, emerging hypotheses suggest that inflammatory processes affecting the visual system may also disrupt broader neural networks, including those regulated by neurotransmitters (17–19). In particular, dopamine—a neurotransmitter involved in various brain functions such as motor control, reward processing, and visual perception—has been implicated in the onset and progression of MS (20, 21). While there is a growing body of literature supporting dopamine role in MS, the precise mechanisms by which it influences the disease progression remain not fully understood (21–24).

This paper presents a system-level, dopamine-based theoretical model to explain the origins of MS, with particular emphasis on its early stages in CIS. Grounded in a focused review of recent literature, the model proposes that demyelination of the optic nerve, as observed in optic neuritis, disrupts dopamine signaling, initiating a cascade of neural changes that contribute to the pathogenesis of MS. Specifically, the model proposes that reduced dopamine release from key dopaminergic nuclei, such as the substantia nigra, leads to altered activity in critical brain regions including the lateral geniculate nucleus (LGN), superior colliculus (SC), and striatum (STR). These disruptions in neural circuits may not only aggravate existing symptoms but also drive broader neurodegenerative processes seen in MS. By focusing on dopamine role during the early stages of CIS and MS, the proposed hypothesis provides a novel framework for understanding the neurobiological mechanisms underlying MS. This approach complements ongoing research into the roles of neurotransmitters in age-related conditions, enhancing understanding of how their imbalances may contribute to the pathogenesis of MS and related disorders (25–27).

## 2 From CIS to MS: the role of optic neuritis, dopamine, and blink reflexes

### 2.1 CIS and MS as system-level disorders

CIS is a neurological condition marked by a single episode of symptoms attributable to inflammation or demyelination within the central nervous system (CNS). It is widely regarded as a precursor to MS, representing a critical window for early diagnosis and potential therapeutic intervention (28, 29). CIS typically presents with focal neurological signs such as optic neuritis, motor or sensory disturbances, or brainstem and cerebellar dysfunction. While these symptoms reflect localized demyelinating damage within the CNS, growing evidence suggests that CIS represents a system-level disorder, involving early disruptions across distributed neural circuits and regulatory networks (6, 9). Diagnosis relies on a thorough clinical evaluation, supported by neuroimaging—particularly magnetic resonance imaging (MRI)—to identify characteristic lesions of demyelination and to exclude alternative causes. MRI findings, including the number, location, and pattern of lesions, are instrumental in assessing the risk of conversion to MS (9, 30).

Not all individuals with CIS progress to MS; however, its presence significantly increases the risk of future disease development. Predictive factors for conversion include the extent of CNS lesions on MRI, recurrence of clinical episodes, and the presence of specific immunological biomarkers (29, 31). Early initiation of disease-modifying therapies following CIS has been associated with reduced disease activity, delayed progression, and improved long-term outcomes. Continuous monitoring and follow-up are essential to track disease evolution, evaluate treatment efficacy, and adapt management strategies in response to clinical or radiological changes. This proactive approach ensures timely intervention and may help mitigate the long-term impact of MS (32, 33).

MS is a chronic autoimmune disorder characterized by inflammation, demyelination, and neurodegeneration within the CNS. Focal lesions in the SC, periventricular cortex (PVC), and SpC characterize MS pathology, disrupting normal neurological functioning and producing diverse clinical symptoms (34–37). This widespread pathology underscores the *system-level* nature of MS, affecting multiple neural circuits and functions across the brain and SpC. The SC, located in the midbrain, plays a key role in visual processing and the coordination of eye movements. Lesions in this area can lead to visual disturbances, such as diplopia (double vision) and impaired eye movements, further highlighting how MS affects complex, interconnected brain systems (38, 39). The SpC serves as the main communication pathway between the brain and the rest of the body. Lesions in the SpC impair motor and sensory functions, leading to weakness, spasticity, numbness, and coordination difficulties (40). MRI plays a crucial role in detecting and characterizing lesions in these widespread regions. Advanced imaging techniques, such as T1-weighted and T2-weighted MRI, along with gadolinium-based contrast agents, allow for detailed visualization of lesions in the SC, SpC, and other affected areas (40, 41). The occurrence of lesions in multiple regions of the CNS—including both visual processing pathways and motor-sensory tracts—illustrates the extensive nature of MS pathology. These lesions contribute to the diverse array of symptoms experienced by individuals with MS and highlight the importance of a system-level approach to diagnosis and management.

### 2.2 Optic neuritis and dopamine in the transition from CIS to MS

The progression from CIS to MS involves a complex, multifactorial cascade, including immune cell activation, inflammatory infiltration, and the influence of both genetic predispositions and environmental exposures (9, 42, 43). Despite advances in imaging and immunological profiling, the exact molecular mechanisms underlying this transition remain incompletely understood and appear to vary significantly across individuals (29). Several studies investigating the transition from CIS to MS have identified key clinical predictors of progression, including the presence of multiple lesions in the brain or SpC. Additionally, detecting oligoclonal bands in the cerebrospinal fluid—a marker of immune activation—strongly correlates with a higher risk of conversion to MS (29, 44). MRI plays a critical role in predicting the likelihood of progression from CIS to MS (45, 46). Studies have demonstrated that the appearance of new or enlarging T2-weighted lesions within the first year following a CIS event significantly elevates the risk of developing MS (47, 48). Similarly, the presence of gadolinium-enhancing lesions at the time of CIS has been shown to be a strong predictor of conversion (28).

Optic neuritis, an acute inflammation of the optic nerve, frequently represents the first clinical manifestation of CIS. Its occurrence in CIS offers valuable insights into the early pathophysiological processes involved in MS development (10, 15, 16). Clinically, optic neuritis often presents as sudden vision loss or visual disturbances, indicative of a localized episode of CNS demyelination. Longitudinal analyses have shown that individuals presenting with optic neuritis as an initial symptom exhibit a heightened likelihood of progressing to clinical MS within a defined timeframe (49, 50).

Dopamine, traditionally linked to reward processing and motor control, also regulates immune responses (51). Altered dopaminergic signaling contributes to neuroinflammation and demyelination. Dopaminergic pathways likely influence CIS pathophysiology. Notably, expression levels of D3-dopamine receptor (DR) and D5-DR mRNA correlate with the risk of conversion to MS within 12 months, suggesting a potential biomarker function and a pathogenic role for dopaminergic signaling in early MS development (20). Functional MRI studies also reveal altered activation of the left putamen—an area rich in dopaminergic input—during attentional tasks in CIS patients, pointing toward early dopaminergic dysfunction in the basal ganglia (52). Together, these findings support the hypothesis that dopaminergic modulation may influence both immune and neural mechanisms in CIS and warrant further investigation into dopaminergic targets for early intervention.

Lending further support to an intrinsic alteration of the dopaminergic system in MS is direct biochemical evidence from cerebrospinal fluid (CSF) studies. Reports in MS patients have documented altered levels of dopamine metabolites, such as homovanillic acid (HVA), in the CSF (53). These findings provide tangible proof of dysfunctional dopamine turnover within the central nervous system, confirming that the hypothesis of a dopamine imbalance is not merely based on indirect mechanisms or animal models but rests on solid biochemical foundations observed in humans. These data reinforce the idea that dopaminergic dysfunction is a fundamental and early feature of MS pathology, warranting consideration as a key factor in the transition from CIS to definite MS.

Altered dopaminergic signaling contributes to neuroinflammation and demyelination, two hallmarks of MS pathology. Studies report findings suggestive of dopaminergic dysfunction and altered signaling in MS patients and other people with neurological disorders, particularly in regions such as the basal ganglia and substantia nigra (54, 55). These reductions correlate with increased inflammatory markers and greater tissue damage. Furthermore, dopamine depletion correlates with dysregulated immune responses, marked by increased pro-inflammatory cytokines and weakened regulatory control (20, 56). This imbalance fosters an environment conducive to demyelination and neurodegeneration. In the CNS, such inflammation-mediated damage to the myelin sheath impairs neuronal communication and contributes to the clinical manifestations of MS (57). Dopamine also directly influences immune cell function. It modulates T cell activation, antigen presentation, and cytokine release (20). Reduced dopaminergic signaling may therefore lead to exaggerated immune responses and a breakdown in self-tolerance, accelerating CNS damage.

Beyond its classical roles in motor control and reward, dopamine is a critical modulator of synaptic plasticity, including long-term potentiation (LTP) and depression (LTD), which are fundamental for learning, memory, and adaptive reorganization. While this function is extensively studied in the context of Parkinson's disease (54, 58), it is equally relevant to MS. In the MS brain, inflammatory and neurodegenerative damage to key dopaminergic projections, such as those to the frontal cortex and striatum, could directly impair the neuroplastic mechanisms essential for functional recovery following a relapse and for cognitive adaptation.

The precise origin of this dopaminergic impairment in MS is a subject of ongoing investigation. It remains to be fully clarified whether altered dopamine signaling is primarily a *consequence of inflammation-driven changes* (e.g., through cytokine-mediated effects on dopamine metabolism and receptor function) or if it represents a *precocious, independent parallel feature* of the disease. However, both scenarios strongly position the dopaminergic system as a compelling target for pharmacological interventions, offering potential as both a symptomatic and a disease-modifying strategy.

Furthermore, the interplay between dopamine and the immune system is bidirectional and more complex than a simple CNS-to-immune signaling axis. As demonstrated by the foundational work of Cosentino, Levite, and others, *the immune system itself is a source of dopamine* (20). Immune cells, including lymphocytes, macrophages, and dendritic cells, can synthesize, release, and respond to dopamine, creating complex autocrine and paracrine feedback loops that can either suppress or promote inflammation depending on the context. This perspective suggests that dopamine dysregulation in MS is a systemic phenomenon, where central nervous system pathology and peripheral immune activation are mutually reinforcing. Therefore, while our model posits that optic neuritis can act as a critical early *trigger* or *amplifier* of this dysfunction, this cascade likely occurs within the broader context of a pre-existing or concurrent systemic neuro-immune imbalance.

### 2.3 The role of blink reflexes

Monitoring blink reflexes (BR) could also serve as a valuable strategy for understanding the transition from CIS to MS, offering insights into the neurophysiological changes that accompany disease progression (59–61). Stimuli from various sensory modalities can elicit reflex blinking as a protective mechanism for the eyes. BR consists of stimulus-triggered responses in the orbicularis oculi muscle, leading to eye closure, similar to spontaneous or voluntary blinks. In clinical practice, the actual movement of the eyelids is often not the primary focus. The most commonly used stimulus for eliciting the BR is electrical stimulation of the supraorbital nerve, which triggers the *trigeminal blink reflex (TBR)* (62–64). Electrical stimuli applied to limb nerves can also induce the blink reflex, known as the somatosensory blink reflex (65, 66), which is closely associated with the *Hand Blink Reflex (HBR)*. Recording bilateral BR responses to unilateral stimulation is a valuable diagnostic tool, as it helps assess potential dysfunctions in the afferent or efferent pathways of the reflex arc. Electrophysiological methods for MS assessment, such as the BR, are typically non-invasive, quick to apply, and cost-effective, providing objective numerical values for detecting dissemination in time and space (60). The evoked BR proves useful in assessing MS-related nervous system damage, as it can reveal “silent” brainstem lesions—areas of demyelination that do not produce overt clinical symptoms but indicate disease progression (59, 67).

Reflexes like the TBR offer valuable insights into how MS affects neural circuits. The TBR, an involuntary blink triggered by stimulation of the ophthalmic branch of the trigeminal nerve, is often disrupted in MS due to demyelination along the trigeminal pathway. Monitoring these changes aids both diagnosis and disease tracking, allowing clinicians to adjust treatment strategies accordingly (61, 68). The TBR consists of two main components. The first is a short-latency, ipsilateral response (R1), mediated by a monosynaptic pathway from the trigeminal sensory nucleus to the facial motor nucleus. The second is a longer-latency, bilateral response (R2), involving a polysynaptic route through interneurons projecting to both facial nuclei. In MS, demyelinating lesions in the brainstem can disrupt these circuits, particularly descending modulatory pathways. This disruption often leads to abnormal excitability, reflected as asymmetries in the amplitude or latency of R1 and R2 responses. Such asymmetries serve as indicators of neural dysfunction and damage. Characterizing these components helps evaluate brainstem involvement in MS. Patients with TBR hyperexcitability often show latency shifts and increased response amplitudes on at least one side. Interestingly, these patients tend to have lower disability scores and reduced tissue loss compared to those with pure latency abnormalities (55, 69). TBR hyperexcitability likely reflects disrupted inhibitory control within trigeminal reflex circuits, shaped by underlying inflammation and demyelination. As such, blink reflex excitability may act as a non-invasive biomarker of early neural dysfunction in MS, with potential utility for both disease monitoring and treatment assessment.

The HBR is a growing focus in MS research (64, 68–71). Unlike the TBR, which involves facial stimulation, the HBR varies with the hand proximity to the face, reflecting peripersonal space representation. Abnormal HBR responses in MS patients indicate brainstem dysfunction, a strong predictor of future disability. We recently applied machine learning to analyze both TBR and HBR in relapsing-remitting MS patients and healthy controls (69, 71). Using features from these reflexes, two AdaBoost classifiers distinguished MS patients from controls with high accuracy, matching or exceeding clinical assessments. This work underscores machine learning potential to probe brainstem function in MS and supports HBR as a promising tool for early diagnosis and brainstem integrity evaluation.

Together, these findings highlight the diagnostic and monitoring value of reflex excitability assessments, especially TBR and HBR. These reflexes reveal distinct dysfunction patterns within trigeminal and brainstem circuits, enhancing understanding of MS pathology and offering potential biomarkers for clinical management.

Direct neuropathological or functional evidence explicitly linking dopaminergic circuit lesions to HBR and TBR dysfunction is currently lacking. The hypothesis presented here is formulated to address this specific knowledge gap by building upon a strong anatomical and functional foundation: the SC, a key node in the proposed circuit, receives significant dopaminergic input from the substantia nigra pars compacta (SNc) and, in turn, plays a crucial modulatory role in brainstem reflex circuits, including those governing the blink reflexes (see Section 3). Dysfunction within this *nigro-collicular dopaminergic pathway* is therefore posited as a plausible and specific mechanism that could directly lead to the aberrant reflex excitability observed in MS patients, moving beyond a general association with “brainstem lesions.” Crucially, this proposed link represents a core, *testable prediction* of the system-level model proposed here. Validation would require future studies combining functional neuroimaging of the dopamine system (e.g., dopamine transporter PET imaging) with electrophysiological assessments of TBR and HBR in patients with CIS. Such work would be essential for pinpointing a specific neurochemical pathway, thereby offering a more precise target for future therapeutic interventions (cf., Section 4.1).

## 3 From CIS to MS: a dopamine-based systems-level hypothesis

### 3.1 Model architecture

**Figure 1:**
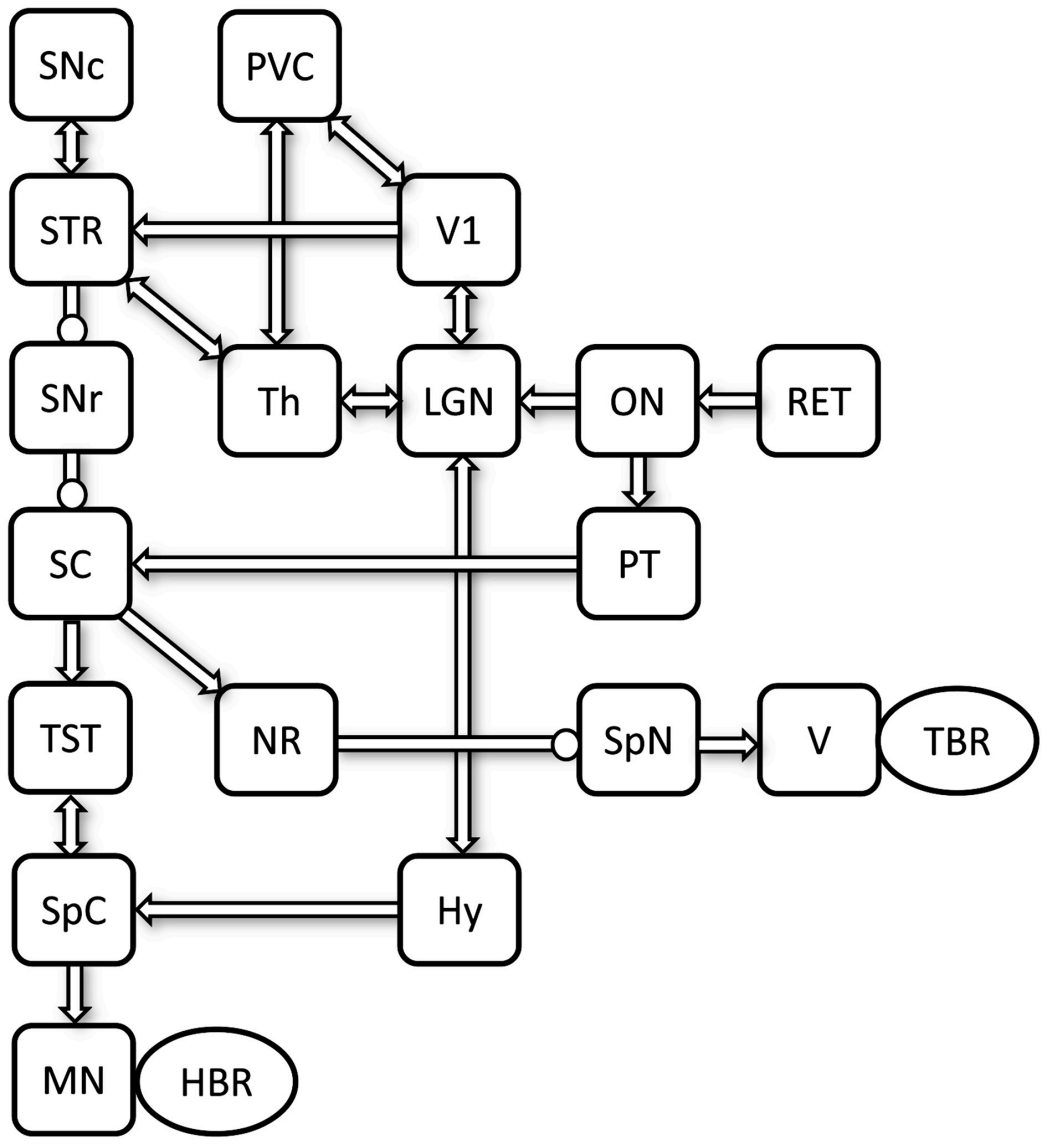
Schematic representation of the proposed visual-dopaminergic circuit involved in early MS progression. Excitatory connections are indicated by arrowheads, whereas inhibitory connections are marked with circles. Hy, hypothalamus; LGN, lateral geniculate nucleus; MN, median nerve; NR, nucleus raphe; ON, optic nerve; PVC, periventricular cortex; PT, pretectum; RET, retina; SC, superior colliculus; SNc, substantia nigra pars compacta; SNr, substantia nigra pars reticulata; SpC, spinal cord; SpN, spinal trigeminal nucleus; STR, striatum; Th, thalamus; TST, tectospinal tract; V, trigeminal nerve; V1, primary visual cortex.

As outlined in the previous section, the progression from CIS to MS reflects a complex interaction of mechanisms, including optic neuritis, dopaminergic imbalance, and disruptions in brainstem reflex pathways. Yet, the precise nature of their interplay and causal dynamics remains unclear. This article introduces a novel system-level hypothesis that implicates a specific neural circuit in driving this transition. The model integrates multiple brain regions into a cohesive network, hypothesized to play a central role in mediating disease progression (Figure 1). The proposed hypothesis posits that disease progression begins with optic neuritis, a frequent early manifestation of CIS, which disrupts dopaminergic regulatory mechanisms. This dysregulation may exacerbate neuroinflammatory responses and impair brainstem-mediated protective reflexes, such as the TBR and HBR, thereby facilitating pathological propagation and accelerating neurodegenerative processes associated with MS.

The optic nerve (ON), which transmits visual input from the retina (RET) to the brain, is commonly affected in optic neuritis (72). ON sends feedforward projections to both the pretectum (PT) (73) and the LGN. PT, which is involved in processing visual input and regulating the pupillary light reflex, projects to the SC, a critical center for visual integration (74). The LGN processes visual information received from the ON and relays it bidirectionally to the primary visual cortex (V1), playing a crucial role in visual perception (74). V1 connects bidirectionally with PVC and the striatum (STR). Additionally, the LGN connects bidirectionally to the hypothalamus (Hy), which influences SpC activity, forming another key relay in visual and dopaminergic signaling. Both the LGN and PVC also communicate with the thalamus (Th), further integrating sensory and dopaminergic inputs (75). The STR, a core input hub of the basal ganglia, receives feedforward input from V1 and connects bidirectionally with Th (58, 74). It is closely linked to the SNc, a primary dopamine-producing nucleus that modulates basal ganglia activity (76). The STR also sends inhibitory projections to the substantia nigra pars reticulata (SNr), which in turn inhibits SC activity (77).

SC function depends on two main pathways—one from the ON–PT circuit and another from the ON–LGN–Th–STR–SNr circuit, where dopamine plays a stronger role. Two distinct pathways then emerge from the SC, each driving a different reflex arc: the TBR and the HBR. In the first pathway, the SC modulates the TBR via projections to the nucleus raphe (NR), which provides inhibitory input to the spinal trigeminal nucleus (SpN). The SpN then communicates with the trigeminal nerve (V), regulating the TBR (77). In the second pathway, the SC sends feedforward projections to the tectospinal tract (TST), which descends through the SpC and controls motor functions (78). The SpC receives inputs from both the TST and the Hy. The SpC then projects to the median nerve (MN), enabling motor and sensory functions involved in the HBR. This model outlines the key neural pathways where disruptions in visual and dopaminergic processing may drive the transition from CIS to MS. Next section explores potential dysfunctions within this framework that contribute to MS pathogenesis.

### 3.2 How CIS becomes MS

This section outlines the system-level neural mechanisms that may drive the progression from CIS to MS. The transition from CIS to MS involves a complex cascade of neural dysfunctions that converge on two distinct, yet potentially overlapping, pathways observed in MS patients: impairments in the TBR and/or the HBR (69). Clinically, CIS often presents with focal symptoms such as optic neuritis, which reflects early inflammatory damage to the central nervous system and marks a critical step toward MS development. Specifically, chronic inflammation of the ON—which consists of axons originating from retinal ganglion cells and transmitting visual information to the brain—leads to vision loss caused by inflammatory swelling and demyelination (79, 80). Emerging evidence links ON inflammation to altered dopamine signaling, although the exact mechanisms remain unclear. Retinal ganglion cells in both On and Off visual pathways may play a role in modulating this relationship (81). Optic neuritis decreases input to PT and LGN, which indirectly disrupts dopaminergic nuclei activity. The LGN projects to STR and SNc via thalamic connections; studies show that reduced input to these regions lowers dopamine release. In turn, diminished dopamine levels contribute to demyelination, linking neurotransmitter dysregulation with inflammatory pathology (21, 82).

Lower dopamine receptor activation in the STR limits inhibition of the SNr, decreasing its inhibitory output and thereby reducing activity in lateral and rostral SC neurons. SC activation further declines due to reduced input from the PT. This loss of excitatory drive from the SC to tonically active neurons in the NR lessens inhibition of trigeminal responsiveness via the spinal trigeminal nucleus (SpN-V) circuit, causing hyperexcitability of the TBR (83–85). At the same time, SC dysfunction could impair the TST, weakening its signaling to the SpC. The LGN also projects to the Hy, a dopaminergic nucleus, where reduced input lowers dopamine release. This exacerbates inflammation, demyelination, and further decreases SpC activation. As a result, spinal cord function deteriorates, impacting the MN and ultimately altering the HBR (70, 86–88). Together, these interconnected disruptions link early focal inflammation in CIS with broader system-level dysfunctions, promoting the progression to full MS pathology through combined neuroinflammatory and dopaminergic mechanisms.

## 4 Clinical relevance of the system-level hypothesis

### 4.1 Implications for early diagnosis and system-level therapies

As discussed in Section 2.3, extensive literature supports the use of functional assessments of reflex pathways, such as the TBR and HBR, as non-invasive tools for monitoring neural circuit integrity and disease progression (61, 69, 89). The hypothesis proposed in this work establishes a direct link between the neural mechanisms underlying dysfunctions in TBR and HBR, thereby extending the diagnostic and therapeutic potential of reflex-based assessments. In this respect, understanding dopamine-based neural circuit disruptions underlying the progression from CIS to MS offers a valuable framework that aligns with emerging research on the role of neurotransmitter systems in neurodegeneration and age-related neurological disorders (90–92). Clarifying the specific contributions of dopaminergic signaling within key neural circuits (Figure 1) provides critical insight into early MS mechanisms and may guide the development of targeted *system-level* neuroprotective strategies to alter the course of disease progression. Early detection of optic neuritis-related dopamine dysfunction could serve as a biomarker to identify patients at higher risk of developing MS. For instance, reduced dopamine transporter (DAT) availability observed through positron emission tomography (PET) imaging in the visual pathways, or abnormal dopamine metabolite levels in the CSF, may indicate early neurochemical changes associated with demyelination. Changes in DAT activity have been observed in MS patients, including reduced striatal dopamine function (93). These measurable indicators not only reflect underlying neural circuit disruptions but also offer a potential window for early therapeutic intervention. DAT inhibitors have shown potential in reducing neuroinflammation and motor deficits in experimental autoimmune encephalomyelitis (EAE), a mouse model of MS (94, 95). Pharmacological interventions aimed at specific dopamine receptor subtypes—particularly those involved in neuroimmune modulation—may offer therapeutic benefits beyond symptomatic relief, potentially altering the underlying pathophysiology of MS (96).

Additionally, functional MRI (fMRI) studies showing altered activation in dopaminergic regions such as the basal ganglia during visual tasks in patients with optic neuritis could help stratify those with a greater likelihood of converting to MS. Patients with optic neuritis exhibit reduced functional connectivity between key visual processing regions, including area V2, which correlates with the severity of visual impairment. These disruptions suggest early-stage network dysfunction that may contribute to long-term visual and cognitive outcomes (97). Individuals presenting with CIS, including those with optic neuritis as the initial manifestation, display atypical patterns of brain activation during motor tasks. These findings are indicative of compensatory cortical reorganization and neural plasticity, which may play a role in modulating disease progression during the earliest phases of MS (30). Functional alterations in basal ganglia networks following episodes of myelitis suggest a broader role in adaptive reorganization, supporting the hypothesis that similar mechanisms may be engaged in optic neuritis and CIS (98). Together, these findings highlight the utility of fMRI in characterizing early neural adaptations in optic neuritis and underscore the potential of functional imaging biomarkers to inform risk stratification and prognosis in individuals at risk of developing MS.

### 4.2 Implications for non-motor symptoms: fatigue, cognition, and neuropsychiatric features

The implications of a systemic dopaminergic imbalance, as proposed in this hypothesis, extend beyond sensorimotor and reflex pathways to provide a mechanistic framework for understanding the most prevalent and debilitating non-motor symptoms of early MS. In light of the extensive projections of the dopaminergic system, our model may offer a potential explanation for the early emergence of fatigue, cognitive deficits, and neuropsychiatric symptoms. A prime example is MS-related fatigue, a pervasive symptom often disconnected from physical disability. The “dopamine imbalance hypothesis of fatigue” posits that this symptom stems from a dysfunction within the mesocortical and mesolimbic reward pathways, leading to deficits in motivation and effort-cost computation (22). The systemic disruption of dopamine signaling, which our model suggests can be triggered by an initial demyelinating event like optic neuritis, provides a plausible cascade through which these critical reward circuits become compromised, leading to the early and profound fatigue experienced by many patients.

Beyond fatigue, a substantial body of evidence links dopamine dysregulation to the broader neuropsychiatric and cognitive burden of MS. The high prevalence of depression and anxiety, for instance, has long been associated with both neuroinflammation and altered monoaminergic neurotransmission. Furthermore, MS patients exhibit a significantly increased risk of developing psychosis or bipolar disorder, suggesting that dopaminergic disruption in key limbic and cortical circuits can lead to profound alterations in thought processing and mood regulation (99, 100). On the cognitive front, impairments in executive function, attention, and processing speed are hallmark features of the disease. These cognitive domains are critically dependent on intact dopaminergic signaling within the prefrontal cortex and its associated networks (101).

Furthermore, the cognitive impairments seen in MS may be linked to dopamine role in regulating neurovascular coupling (NVC)—the process that matches local blood flow to neural activity. Dopaminergic signaling, particularly through D2/D3 receptors, is known to modulate NVC dynamics in the frontal lobes (102). Given that dysfunctional NVC has been observed in MS and may contribute to cognitive deficits (103, 104), an early dopaminergic deficit could disrupt cognitive function through a dual mechanism: by impairing direct synaptic transmission and by compromising the metabolic and hemodynamic support provided by healthy neurovascular units (105).

Therefore, by viewing early MS through the lens of a developing dopamine imbalance, our model offers a unifying perspective. It connects an initial focal inflammatory insult to a wide spectrum of clinically crucial outcomes—from reflex abnormalities to fatigue and cognitive decline. This expanded framework underscores the potential of dopaminergic pathways not only as a biomarker for risk stratification but also as a promising target for comprehensive therapeutic interventions aimed at alleviating the full constellation of MS symptoms. From a therapeutic standpoint, strategies aimed at restoring dopaminergic balance and modulating neural excitability within the system showed on Figure 1 could slow or prevent the transition to MS. Interventions targeting the optic nerve, dopaminergic nuclei, and downstream circuits may reduce neuroinflammation and protect myelin integrity. A systemic perspective provides a valuable foundation for developing neuromodulation strategies in MS. Circuit-level interventions, guided by systemic models of dysfunction, indeed, could reduce fatigue-related symptoms and improve quality of life in individuals with MS (106). In addition, system-level therapies that integrate sensory processing and motor reflex regulation show promise for slowing neurological decline and improving clinical outcomes (107).

The therapeutic implications of our hypothesis suggest that direct pharmacological modulation of the dopaminergic system could offer clinical benefits. This rationale is supported by previous clinical investigations into dopaminergic agents, such as amantadine and methylphenidate, for the management of MS-related fatigue. Although these trials have yielded mixed results (108, 109), they underscore the long-standing interest in this pathway and support its therapeutic potential. Interestingly, a contemporary and highly relevant link to this mechanism can be found in ozanimod, a recently approved disease-modifying therapy for MS. While primarily acting as a sphingosine-1-phosphate (S1P) receptor modulator, ozanimod also functions as a weak and reversible monoamine oxidase B (MAO-B) inhibitor (110). As MAO-B is a key enzyme in the catabolism of dopamine, its inhibition can increase the synaptic availability and half-life of CNS dopamine. This dual mechanism, while perhaps a secondary effect, provides a compelling connection between a modern, approved MS therapy and the dopaminergic dysfunction highlighted in our model, suggesting that some of its clinical benefits could be partially mediated through this pathway and warranting further investigation into this pharmacological avenue.

## 5 Conclusions and future works

The progression from CIS to MS highlights the critical role of neurotransmitter systems—particularly dopaminergic signaling—in mediating neuroinflammatory and neurodegenerative processes. This perspective aligns with broader mechanisms underlying other age-related neurological disorders (111–113). The modulatory role of dopamine in the CIS-to-MS transition suggests that therapeutic approaches aimed at preserving or restoring dopaminergic balance could help mitigate inflammation and demyelination. Such strategies may hold cross-disease relevance, offering insights applicable to a wider spectrum of neurodegenerative conditions associated with aging (114, 115). Nevertheless, further research is needed to unravel the precise mechanisms by which dopamine and related neurotransmitters interact with immune and neural networks in aging brains susceptible to MS.

### 5.1 The role of dopamine imbalance in progressive multiple sclerosis

While our hypothesis focuses on the initial triggers of MS, its principles can be extended to explain the role of dopamine in the transition to and maintenance of progressive multiple sclerosis (PMS). The pathogenesis of PMS is increasingly viewed not simply as an accumulation of damage, but as a *failure of intrinsic CNS compensatory mechanisms*, including synaptic remodeling, debris clearance, and crucially, remyelination (116–118). A chronic dopaminergic deficit, as proposed here, could be a key factor driving this failure. Emerging evidence suggests that dopamine signaling is directly involved in myelin homeostasis. Dopamine receptors are expressed on oligodendrocyte precursor cells (OPCs), and dopamine signaling has been shown to promote OPC proliferation and differentiation, which are essential for successful remyelination (119–121). Consequently, a sustained reduction in CNS dopamine could directly impair the brain capacity to repair myelin damage, thus contributing to the accumulation of chronic demyelinated lesions seen in PMS.

Furthermore, this chronic dopamine deficit could shape the trajectory of neurodegeneration through a “double-hit” mechanism. Beyond its role in remyelination, dopamine has neuroprotective and immunomodulatory functions. Its absence could exacerbate chronic, smoldering inflammation driven by microglia and contribute to oxidative stress, thereby accelerating neuroaxonal and synaptic loss (51, 122). This framework helps differentiate the role of dopamine across the disease course. In relapsing-remitting MS, dopamine signaling may be acutely disrupted but can partially recover, allowing for periods of CNS compensation. In contrast, in PMS, a chronic and worsening dopamine deficit becomes a critical driver of the progressive phase by undermining the very repair and neuroprotective mechanisms needed to maintain CNS integrity. This sustained imbalance helps explain the insidious accumulation of disability that occurs independently of relapses. In cases of rapidly progressive disease, a particularly severe initial dopaminergic insult or a lower intrinsic reserve for compensation could accelerate this transition, highlighting the importance of dopamine signaling as a potential determinant of disease trajectory from the earliest stages.

The role of specific dopamine receptors in the processes described above presents a translational paradox that underscores the system complexity. Intriguingly, preclinical studies have shown that D2 receptor antagonists, such as haloperidol and risperidone, can promote remyelination in toxin-induced models and ameliorate disease severity in EAE, though the exact mechanisms remain to be fully elucidated (123, 124). These promising findings stand in stark contrast to the results of a recent Phase 2 clinical trial, where the D2 receptor blocker domperidone failed to show overall efficacy in slowing progression in patients with SPMS (125). This discrepancy highlights the context-dependent role of dopamine signaling and the significant challenges in translating findings from animal models to human progressive disease, underscoring the need for more nuanced therapeutic strategies that go beyond simple receptor blockade.

### 5.2 Toward a multifactorial system-level perspective

Beyond dopamine, future studies should investigate how other neurotransmitter systems—such as serotonin, noradrenaline and acetylcholine—intersect with dopaminergic pathways and contribute to the complex neurochemical cascades that drive MS progression. Recent computational and experimental models emphasize the importance of these complex interactions in neurodegenerative diseases (26, 126–128). Additionally, it is essential to consider the roles of lifestyle factors—including diet, physical activity, and stress management—and genetic predispositions in modulating neurotransmitter function and influencing susceptibility to MS and other age-related diseases (129, 130).

Future research should also examine sex-related differences in MS onset, progression, and treatment response. Hormonal and immunological factors may differentially modulate neurotransmitter systems, influencing both disease vulnerability and therapeutic outcomes (131). More broadly, addressing sex-based variation in aging-related neurological disorders is critical for developing targeted interventions that reflect distinct biological and neuroendocrine trajectories (132, 133). Finally, combining explainable machine learning with systems-level computational modeling could offer a powerful approach to improve diagnostic precision and gain deeper insight into the underlying biological mechanisms (69, 134). These methods could uncover hidden patterns in multimodal data, simulate circuit-level dynamics, and identify key modulators of disease progression. Such approaches may enhance predictive accuracy and support biologically grounded, interpretable decision-making in clinical settings.

Together with considerations of sex differences, environmental exposures, and aging, these dimensions contribute to a more comprehensive understanding of disease etiology and open new avenues for personalized prevention and intervention. Advancing knowledge of these *multifactorial influences* remains essential for designing targeted, system-level therapies to preserve neurotransmitter balance, slow neurodegeneration, and support healthy aging (135–139).

## Data Availability

The original contributions presented in the study are included in the article/supplementary material, further inquiries can be directed to the corresponding author.
